# Breaking Up the C Complex Spliceosome Shows Stable Association of Proteins with the Lariat Intron Intermediate

**DOI:** 10.1371/journal.pone.0019061

**Published:** 2011-04-19

**Authors:** Patricia Coltri, Kerstin Effenberger, Robert J. Chalkley, A. L. Burlingame, Melissa S. Jurica

**Affiliations:** 1 Department of Molecular, Cell and Developmental Biology, University of California Santa Cruz, Santa Cruz, California, United States of America; 2 Center for Molecular Biology of RNA, University of California Santa Cruz, Santa Cruz, California, United States of America; 3 Department of Pharmaceutical Chemistry, University of California San Francisco, San Francisco, California, United States of America; Tulane University Health Sciences Center, United States of America

## Abstract

Spliceosome assembly requires several structural rearrangements to position the components of the catalytic core. Many of these rearrangements involve successive strengthening and weakening of different RNA∶RNA and RNA∶proteins interactions within the complex. To gain insight into the organization of the catalytic core of the spliceosome arrested between the two steps of splicing chemistry (C complex), we investigated the effects of exposing C complex to low concentrations of urea. We find that in the presence of 3M urea C complex separates into at least three sub-complexes. One sub-complex contains the 5′exon, another contains the intron-lariat intermediate, and U2/U5/U6 snRNAs likely comprise a third sub-complex. We purified the intron-lariat intermediate sub-complex and identified several proteins, including U2 snRNP and PRP19 complex (NTC) components. The data from our study indicate that U2 snRNP proteins in C complex are more stably associated with the lariat-intron intermediate than the U2 snRNA. The results also suggest a set of candidate proteins that hold the lariat-intron intermediate together in C complex. This information is critical for further interpreting the complex architecture of the mammalian spliceosome.

## Introduction

Pre-mRNA splicing is an essential step in eukaryotic gene regulation. The excision of introns coupled to exon ligation to form mRNA molecules is performed by the spliceosome, a huge macromolecular machinery composed of five snRNPs (U1, U2, U4/U6 and U5 snRNAs associated with proteins) and many additional splicing factors. In total the complex comprises approximately 175 components [1]. Spliceosome assembly at each intron is a dynamic process, and formation of a catalytically competent complex is dependent on rearrangements of many RNA∶RNA, RNA∶protein and protein∶protein interactions [2], [3], [4].

One of many examples of spliceosome dynamics is the extensive and shifting interaction network of U2 snRNP during spliceosome assembly and catalysis. Changes in internal U2 snRNA base pairing, base pairing with the pre-mRNA and U6 snRNA have been demonstrated [5], [6], [7]. U2 snRNA∶protein interactions also evolve during spliceosome assembly. For example, when compared to the isolated 17S U2 snRNP particle, crosslinking of proteins with the U2 snRNA, 5′ splice site and branchpoint regions changed progressively in both A and B complexes [8]. Although far from fully understood, U2 snRNP protein interactions within the snRNP and with other spliceosome components also exist and change during splicing.

Many other interactions (and their changes) within the spliceosome have been described. However, given the numerous players and large combinatorial potential for interactions, a clear picture of the arrangement of the spliceosome's many components and their intermolecular associations would be helpful, but is not currently available. For example, little is known about the dynamics of the Prp19 complex (NTC) and its associated proteins, which join the spliceosome at some point during B complex formation [9]. NTC plays roles in modulating the interactions of both U6 and U5 snRNAs with pre-mRNA during spliceosome activation [10], [11]. Genetic evidence supports a role for the core NTC protein Isy1 in promoting first step fidelity in conjunction with the ATPase Prp16 [12]. However, the interactions that mediate these functions are not known. Direct interactions between NTC core proteins and other spliceosome proteins have not been characterized, although NTC-associated proteins Cwc21 and Cwc2 have been shown to interact with Prp8 and U6 snRNAs, respectively [13].

Working toward the goal of characterizing the interactions within the catalytic core of the spliceosome that lead to specific positioning of pre-mRNA and splicing, we examined the stability of interactions of pre-mRNA with spliceosomal core components after the first step of chemistry (i.e. in C complex spliceosomes). We find that the 5′ exon and lariat intermediate disassociate in the presence of 3M urea, but are not entirely stripped of binding proteins. Closer examination of the lariat intermediate reveals that several U2 snRNP proteins, NTC proteins and other spliceosome proteins remain associated. Our results address the hierarchy of complex protein-RNA interactions in C complex, which will be important for modeling spliceosome structures in the future.

## Results

### The 5′ exon and lariat-intron intermediate in C complex spliceosomes disassociate in 3M urea

In order to assess the stability of core C complex spliceosomes we exposed purified complexes to increasing concentrations of urea and examined their sedimentation behavior in glycerol velocity gradients. As previously described [14] we first assembled C complex spliceosomes in HeLa nuclear extracts on a pre-mRNA splicing substrate that harbors an AG→GG mutation at the 3′ splice site. This splicing substrate goes through the first step of chemistry, yielding a cleaved 5′ exon and lariat-intron intermediate, but the second step is blocked due to the 3′ splice site mutation, and a population of C complex spliceosomes accumulates. We isolated the splicing complexes by an MS2∶MBP affinity tag located in the intron of the pre-mRNA under native conditions. We then treated the isolated complexes with varying amounts of urea before sedimentation in a linear (10–30%) glycerol gradient. To follow the complexes, we examined the radiolabeled pre-mRNA splicing intermediates extracted from gradient fractions by denaturing gel analysis.

**Figure 1:**
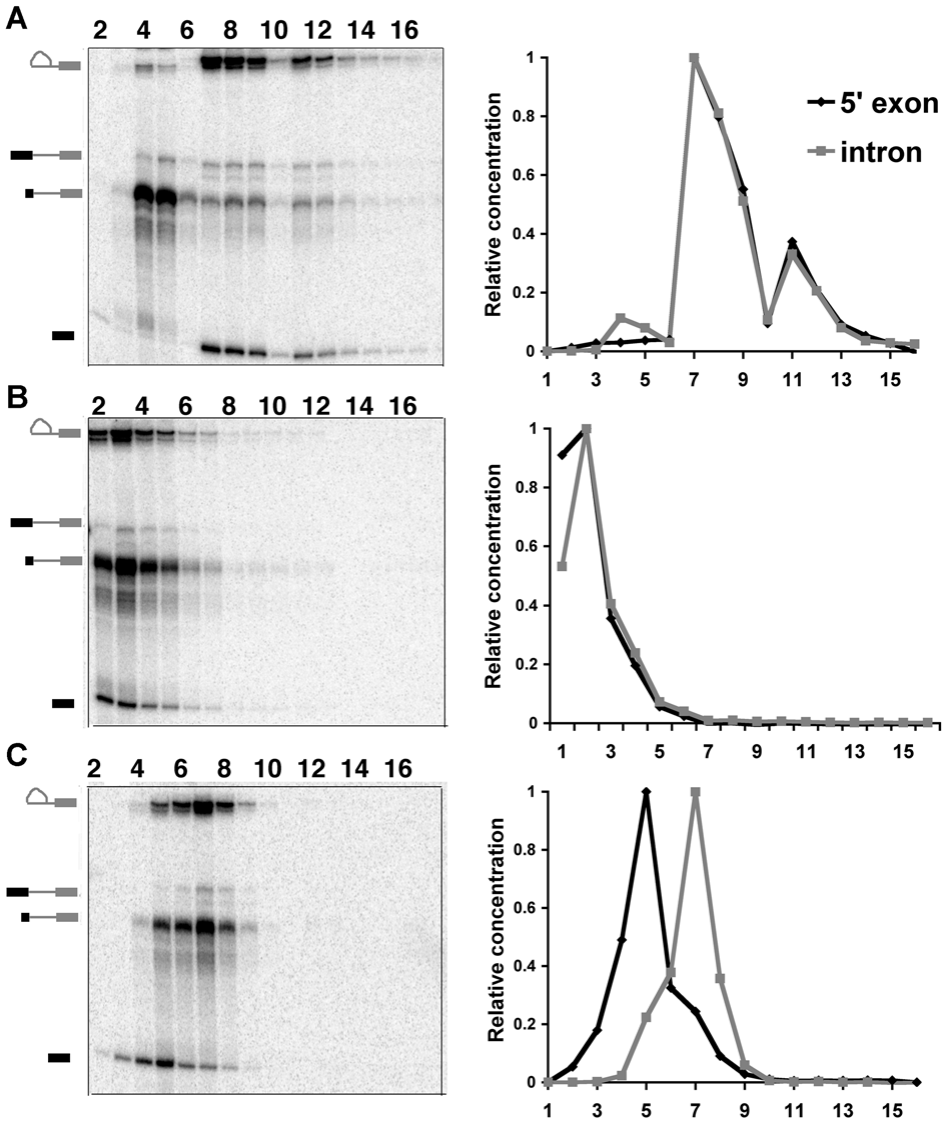
Glycerol gradient profiles of purified C complex spliceosomes. All three panels show denaturing gel analysis of RNA from linear (10–30%) glycerol gradient sedimentation of (a) native, (b) phenol-extracted and (c) 3M urea-treated C complex spliceosomes. Fractions numbers from the top to the bottom of the gradient are indicated. Splicing intermediates are indicated on the left. From top to bottom: lariat-intron intermediate, pre-mRNA, digested pre-mRNA and 5′ exon. The digested pre-mRNA arises from RNase H mediated cleavage of substrate that did not incorporated into C complex during spliceosome assemble as detailed in [14]. The panels on the right show the normalized quantifications of 5′ exon (black) and lariat-intron (grey) intermediates in glycerol gradient fractions.

With no urea treatment, both 5′ exon and lariat-intron intermediates of C complex peak in the same gradient fractions (fraction 7–8) in equimolar amounts, which is expected for the intact complex [14] (Figure 1A). In contrast, after completely disrupting the complex by phenol extraction to obtain protein-free RNA, we find that both the 5′ exon and lariat-intron intermediate peak at the top gradient in fraction 2 (Figure 1B). Treating complexes with 1M urea prior to gradient fractionation did not alter the mobility of the splicing intermediates, suggesting that they remain together in a complex (Figure S1). However, when we incubated the complexes in 3M urea, we found that the 5′ exon and lariat-intron intermediate no longer sediment in the same fractions. Instead the 5′ exon peak shifted two fractions upward in the gradient to fraction 5, while the lariat-intron intermediate was concentrated between fractions 6 and 7 (Figure 1C). Based on these results, we hypothesized that 3M urea disrupts key interactions stabilizing C complex and leads to its separation into at least two sub-complexes, one of which contains the 5′ exon and other containing the lariat-intron intermediate. It is possible that some proteins disassociated from the splicing intermediates with the urea treatment. However, because neither RNA species moved to the top of the gradient like protein-free RNA, we infer that some spliceosome components remain associated with both the 5′ exon and the lariat-intron intermediate.

**Figure 2:**
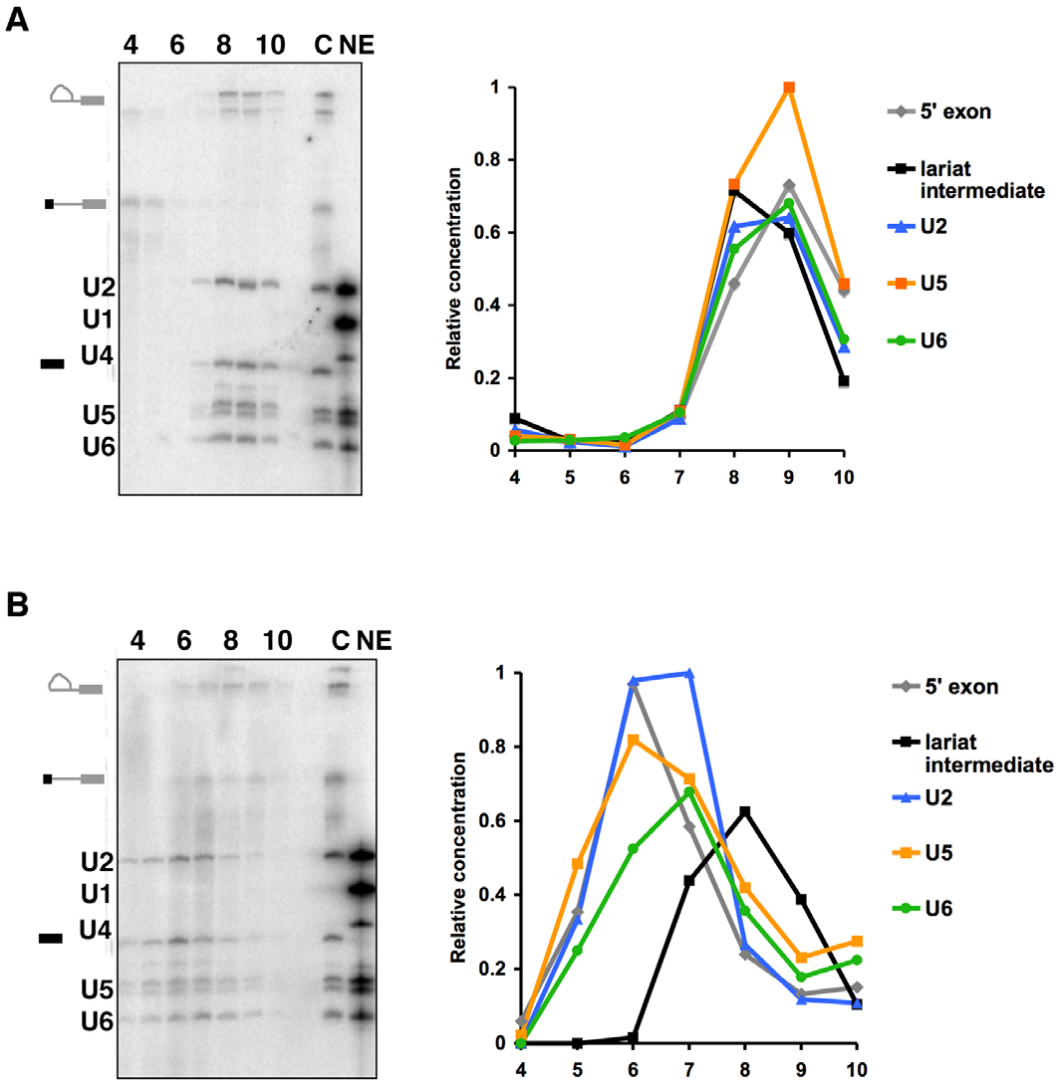
Glycerol gradient profiles of spliceosomal U snRNAs associated with purified C complex spliceosomes. The two panels show northern blots using probes against U1, U2, U4, U5, U6 snRNAs for (a) native and (b) 3M urea-treated C complex spliceosomes fractionated on glycerol gradients. Fractions numbers from the top to the bottom of the gradient are indicated. Splicing intermediates and snRNAs are indicated on the left. The panels on the right show the quantifications of 5′ exon (grey), lariat-intron intermediate (black) and the 3 snRNAs found in C complex (U2, blue; U5, green; U6, orange). C is native C complex; NE is nuclear extract control.

To explore this hypothesis, we investigated the profile of spliceosomal snRNAs from repeated gradient fractionation of native and 3M urea treated C complexes. Northern analysis for the five U snRNAs, show that U2, U5 and U6 snRNAs co-migrate with both splicing intermediates in native C complex, as previously demonstrated [14] (Figure 2A). With 3M urea treatment, however, all three snRNAs follow the 5′ exon shift and peak together three fractions higher in the gradient (Figure 2B). Although this result is suggestive of a complex containing the 5′ exon and snRNAs, the data do not differentiate between this possibility and the possibility of two or more separate snRNA sub-complexes.

### Isolation of separate intron-intermediate and 5′ exon complexes

**Figure 3:**
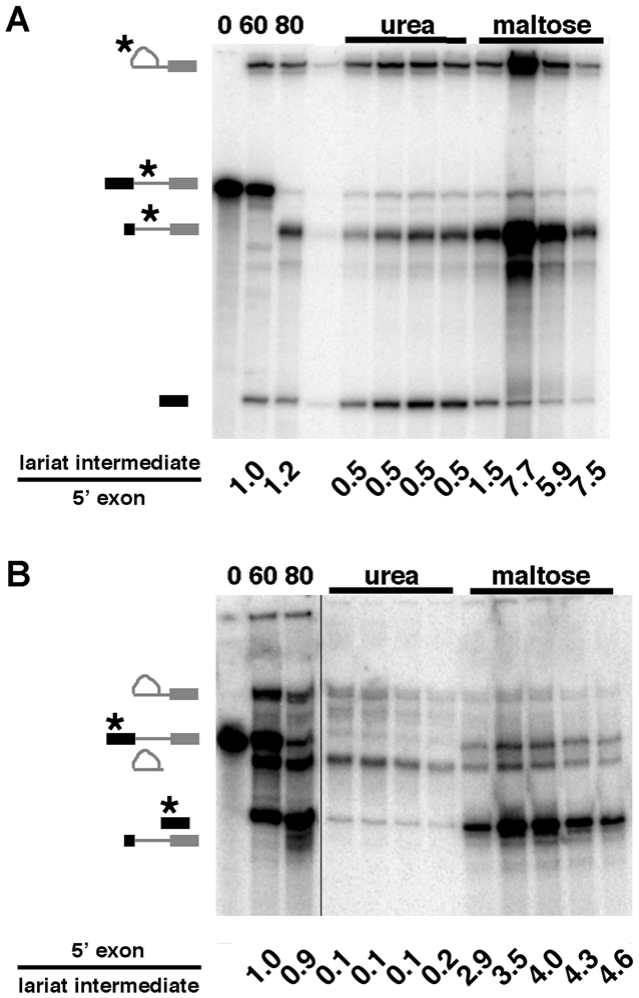
Purification of intron-lariat intermediate and 5′ exon subcomplexes. (a) Denaturing gel analysis of sample fractions taken during purification with an intron-tagged pre-mRNA substrate. From left to right: Indicated time points of splicing reaction in minutes, fractions from 3M urea wash and maltose eluate. Splicing intermediates and other RNA species are indicated on the left. From top to bottom: lariat-intron intermediate, pre-mRNA, digested pre-mRNA that did not assemble into splicing complexes and 5′ exon. The asterisk indicates species that contain the MS2∶MBP affinity tag. Quantified below each lane is the relative ratio lariat-intron intermediate to 5′ exon. (b) The same as (a) except that the affinity tag is located in the 5′ exon of the pre-mRNA substrate. In this case the splicing intermediates and other RNA species are (from top to bottom) lariat-intron intermediate, pre-mRNA, 3′ end chew back product of lariat-intron intermediate, 5′ exon and digested pre-mRNA that did not assemble into splicing complexes. The quantification below is the relative ratio of 5′ exon to lariat-intermediate species.

In order to examine the complexes associated with the splicing intermediates more closely, we sought to isolate them separately. We altered our C complex purification protocol to add a 3M urea wash step at the point where C complex is bound to amylose resin just prior to affinity elution with maltose (Figure 3). As predicted by the glycerol gradient analysis, we find that most of the 5′ exon elutes in the urea fractions, while the MS2∶MBP tagged lariat-intron intermediate is largely retained on the column. Subsequent addition of maltose (3–10 mM) elutes the lariat-intron intermediate separately (Figure 3A). When we move the MS2∶MBP tag from the intron to the 5′ exon of the splicing substrate, we obtain the reverse situation: lariat-intron intermediate elutes with the urea wash, while the 5′ exon is retained until maltose elution (Figure 3B). These results show that we can enrich for either RNA species separately. It also supports our hypothesis that 3M urea treatment disrupts interactions that hold the 5′ exon and lariat-intron intermediate together in C complex after the first step of chemistry.

**Figure 4:**
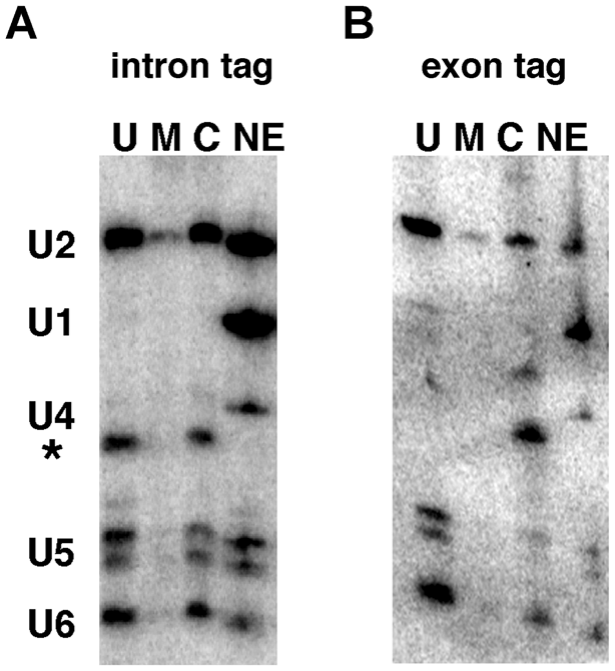
snRNAs association with splicing intermediates after 3M urea treatment. Northern analysis probing for U1, U2, U4, U5 and U6 snRNAs from purified C complex spliceosomes using (a) an intron-tagged or (b) 5′ exon-tagged pre-mRNA substrate. NE is nuclear extract control. C is native C complex. U is 3M urea wash of C complex bound to amylose resin. M is the subsequent elution with maltose. The asterisk indicates the signal from the radiolabeled 5′ exon.

In order to address the potential association of snRNPs with the 5′ exon, we performed northern blots for the spliceosomal U snRNAs in peak fractions from urea and maltose elution of C complex assembled on pre-mRNA substrate with the MS2∶MBP tag in either the intron or 5′ exon. With either tagged substrate, we find the U2, U5 and U6 snRNAs in the urea elution (Figure 4). This result indicates that the U snRNAs are not stably associated with either 5′ exon or lariat-intron intermediate after urea treatment. It thus appears that 3M urea also disrupts interactions stabilizing the association of the three U snRNAs with C complex. From these experiments we cannot tell whether the snRNAs remain in association with each other, although it may be indicated by their co-sedimentation in glycerol gradients even after urea treatment (see Figure 2). However, this aspect of the urea treatment remains to be explored.

### Several proteins remain associated with urea-washed lariat-intron intermediate

We next questioned whether proteins remain bound to the lariat-intron intermediate after urea treatment. Although it is possible that 3M urea treatment may completely disrupt C complex, the fact that both the 5′ exon and lariat-intron intermediate sediment significantly farther in glycerol gradients relative to their naked RNA counterparts indicates that some spliceosome components are still in complex. Furthermore, our ability to purify the urea-washed splicing intermediates indicates that at least the MS2∶MBP protein remains bound to the RNA. To determine which proteins disassociate from the lariat intermediate with urea treatment and which proteins remain bound, we pooled fractions from the urea wash and maltose elution of C complex assembled on a lariat-intron tagged substrate. We then subjected those samples to protein identification by mass spectrometry (MS). The results of this analysis are shown in Table 1.

**Table 1:** Mass spectrometry analysis of urea and maltose eluates from C complex purification.

UniProt accession	Protein	S.c.	kDa	Urea	Maltose
***Sm proteins:***					
P14678	**SmB/B′**	Smb1p	*24*	**2**	
P62314	**SmD1**	Smd1p	*13*	**3**	**2**
P62316	**SmD2**	Smd2p	*13*	**3**	
P62318	**SmD3**	Smd3p	*14*	**3**	**2**
P62304	**SmE**	Sme1p	*11*	**1**	
P62306	**SmF**	Smf1p	*10*	**2**	
***U2 snRNP:***					
P09661	**U2-A′**	Lea1p	*28*	**7**	**5**
P08579	**U2-B″**	Msl1p	*25*	**4**	**4**
Q15459	**SF3a120**	Prp21p	*88*	**2**	**4**
O75533	**SF3b155**	Hsh155p	*146*		**22**
Q13435	**SF3b150**	Cus1p	*98*		**11**
Q15393	**SF3b130**	Rse1p	*135*	**4**	**15**
Q15427	**SF3b50**	Hsh49p	*44*		**1**
Q7RTV0	**SF3b14**	Rds3p	*12*		**1**
***U5 snRNP:***					
Q6P2Q9	**PRPF8**	Prp8p	*273*	**77**	**25**
O75643	**U5–200 kD**	Brr2p	*244*	**56**	**18**
Q15029	**U5–116 kD**	Snu114p	*109*	**13**	**10**
Q96DI7	**U5–40 kD**		*39*	**2**	**3**
***Recruited to B complex:***					
Q9HCG8	**CWC22**	Cwc22p	*105*	**3**	**3**
P23246	**PSF**		*76*	**13**	**1**
***NTC:***					
Q9UMS4	**PRP19**	Prp19p	*55*	**26**	**7**
Q99459	**CDC5L**	Cef1p	*92*	**7**	**9**
O75934	**SPF27**	Snt309p	*26*	**9**	**1**
O43660	**PLRG1**	Prp46p	*57*	**1**	**7**
***NTC associated:***					
Q13573	**SKIP**	Prp45p	*61*	**7**	**2**
Q9BZJ0	**CRNL1**	Clf1p	*100*	**6**	**6**
Q9HCS7	**SYF1**	Syf1p	*100*	**2**	**13**
Q9ULR0	**ISY1**	Isy1p	*32*		**6**
Q9NW64	**RBM22**	Ecm2p	*47*		**6**
Q9UNP9	**PPIE**		*33*		**1**
Q9Y3C6	**PPIL1**		*18*		**1**
***Second step factors:***					
O95391	**SLU7**	Slu7p	*68*	**2**	
O60508	**PRP17**	Prp17p	*65*	**2**	**3**
Q14562	**DHX8**	Prp22p	*139*	**6**	**13**
***Recruited to C complex:***					
O60306	**AQR**		*171*	**5**	**52**
Q9Y314	**NOSIP**		*33*		**3**
Q96BP3	**PPWD1**		*74*	**2**	
Q9UJV9	**DDX41**		*70*	**2**	
Q9H2H8	**PPIL3**		*18*	**1**	
O95926	**GCIP-IP**	Syf2p	*28*	**1**	
***Exon junction complex:***					
P38919	**eIF4AIII**		*47*	**4**	**8**
***hnRNP/RNA binding proteins *** * ***:***					
Q13151	**hnRNPA0**		*31*		**3**
P09651	**hnRNPA1**		*39*		**5**
P22626	**hnRNPA2/B1**		*37*		**2**
P07910	**hnRNPC**		*33*	**4**	**14**
Q9UKM9	**RALY**		*32*		**1**
P26599	**PTBP1**		*57*		**15**
Q86U42	**PABP2**		*32*	**1**	
Q9H875	**PKRI1**		*21*	**1**	
Q96PU8	**QKI**		*37*		**17**
P43243	**MATR3**		*94*		**17**
Q9Y580	**RBM7**		*30*		**2**
Q15717	**ELAV1**		*36*		**1**

LC-MS/MS analysis of urea and maltose eluate of lariat-intron tagged C complex spliceosome purification. Columns from left to right are the UniProt accession number, human protein name, *S. cerevisiae* homolog name, molecular weight in kDa, number of unique peptides used to identify a given protein in urea and maltose eluates.

* These proteins have been previously shown to associate with pre-mRNA under conditions that do not support splicing.

For several proteins detected from lariat-intron tagged C complex we see a clear enrichment when we compare of the number of unique peptides sequenced in the urea vs. maltose eluate. In cases where the difference between the number of peptides found for each sub-complex was higher than 70% of the total, we considered the protein enriched. For example, we detected 77 unique peptides for the U5 snRNP protein Prp8 in the urea eluate, but only 25 unique Prp8 peptides in the maltose eluate. In contrast, we detected 22 peptides from the U2 snRNP protein SF3b155 in the same maltose eluate, but no SF3b155 peptides in the urea eluate. For some proteins, there is no clear enrichment, an example of which is U2-B″. We detected four U2-B″ peptides in both the urea and maltose eluate. For some of the other C complex proteins, we did not obtain large numbers of peptides, making it difficult to strongly conclude differential representation in the two samples.

In comparing the peptide numbers for the different C complex proteins, there are several clear trends in the data. The number of peptides for U5 snRNP proteins is greater in the urea eluate relative to the maltose eluate, with the exception of the U5 40 kD for which two peptides were in the urea wash sample vs. three in the maltose eluate. In contrast, with the U2 SF3b complex proteins that we detected, there were more peptides in the maltose elution. Sm proteins, which are small and therefore yield fewer peptides, were all enriched in the urea eluate. Whereas a number of Prp19 complex (NTC) and NTC-associated proteins were enriched in the urea eluate or nearly equally represented, several were clearly enriched in the maltose eluate. Second step factors Prp17 and Prp22 were also enriched in the maltose sample, while Slu7 peptides were only found in the urea eluate. Of the six C complex specific proteins that we detected, only two, AQR (IBP160) and NOSIP, yielded peptides enriched in the maltose elution. Finally, peptides that we detected for many RNA-binding proteins, which are likely not specific components of C complex (ex. hnRNP proteins), nearly all were found only in the maltose sample.

**Figure 5:**
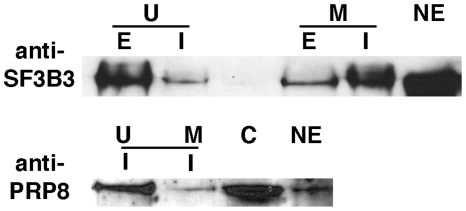
Association of SF3b130 and Prp8 with splicing intermediates after 3M urea treatment. Western analysis of purified C complex spliceosomes using an intron-tagged (I) or 5′ exon-tagged (E) pre-mRNA substrate. NE is nuclear extract control. C is native C complex. U is 3M urea wash of C complex bound to amylose resin. M is the subsequent elution with maltose.

Although MS analysis is not strictly quantitative, there is a strong correlation between the number of peptides identified to a given protein and its amount [15]. Hence, differential peptide numbers in the urea vs. maltose elution for a given protein provides a measure of its association with the tagged lariat-intron intermediate. We tested this idea for two spliceosome proteins by western analysis of the urea and maltose eluates (Figure 5). In agreement with our hypothesis, we observe a significant enrichment of U2 SF3b130 in the maltose eluate of the lariat-intron tagged substrate relative to the urea eluate. This correlates well with the MS results for this protein, which showed 15 peptides in the maltose sample vs. four in the urea sample. In contrast, if the affinity tag is located in the 5′ exon of the pre-mRNA substrate such that lariat-intron intermediate is released during the urea wash, we observe the reverse situation: SF3b130 is enriched in the urea vs. maltose samples. With the U5 snRNP protein Prp8 we see enrichment in the urea eluate relative to the maltose eluate with the intron-tagged pre-mRNA substrate, again consistent with MS results discussed above. These results support our hypothesis that some proteins remain stably associated with the lariat intermediate in 3M urea, while others do not.

## Discussion

The composition and gross structure of C complex have been characterized, however the relative spatial arrangement of spliceosomal components in that structure has not been determined. Years of biochemical and genetic studies have defined several subcomplexes of spliceosomal components and many direct interactions between these components, which provide important clues to their relative positions. However, the multitude of components and dynamic complexity of the spliceosome as it evolves during the splicing cycle leaves much still to learn about the architecture of the complex. In this study we explore a set of stable interactions in C complex spliceosomes. We find several proteins that remain stably bound to the lariat intermediate in the presence of low concentrations of urea, while many other spliceosome components are released with this treatment. While a number of the bound proteins are known to interact with RNA and are likely associated by directly binding the lariat intermediate (ex. MS2∶MBP and hnRNP proteins), it is likely that some of the associated proteins are retained through stable protein-protein interactions. These proteins are good candidates for mediating interactions within C complex that assist in holding both intermediates together. For example, we find the SF3b trimer of the 155, 150 and 130 kD proteins all enriched. Currently there is evidence to support only SF3b150kD interaction with pre-mRNA in C complex [16], [17], and the other SF3b proteins likely remain through their stable interactions with each other and potentially other spliceosome proteins. Although SF3b has been shown to interact directly with U2 snRNA in the U2 snRNP [18], release of U2 snRNA from C complex with the urea treatment is not surprising. During activation of splicing, U2 snRNA becomes extensively base paired with U6 snRNA, which likely requires a change in its association with U2 snRNP proteins. In fact after urea treatment we find U2 migrating with U6 snRNAs on glycerol gradients. It has been recently noted that SF3Bs association with the spliceosome is destabilized during Prp2 mediated spliceosome activation, which may be a reflection of its having “let go” of the U2 snRNA [19], [20].

In addition to the SF3B proteins, several proteins associated with the NTC show striking peptide enrichment with the lariat intron. These include PLRG1 (WD40 motif), SYF1 (HEAT repeat), ISY1 (no obvious motifs) and RBM22 (RRM, zinc finger). Of these, only RBM22 has an RNA-binding motif, making it a potential candidate for directly binding to the lariat-intron intermediate. Alternatively or additionally, these NTC-associated proteins may associate with the lariat-intron intermediate via interactions with SF3B proteins.

Two other proteins that show significant enrichment with the intron are AQR and DHX8 (Prp22), both of which contain helicase motifs. DHX8 is a second step factor and has been shown to be important for exon ligation and mRNA release from the spliceosome in yeast [21], [22], [23]. The yeast homolog Prp22 has been shown to contact the 3′ exon following the second step of chemistry, but no data yet shows a direct interaction with pre-mRNA in C complex. AQR, which joins the spliceosome at or after the first step of chemistry, is also known as intron-binding protein 160 (IBP160) and has been shown to crosslink upstream of the branchpoint sequence [24].

After the first step of splicing it is imperative that the spliceosome retains a tight grip on the splicing intermediates to ensure proper exon ligation. Although several interactions between the pre-mRNA and snRNAs or proteins have been demonstrated, our understanding of this critical function of the spliceosome is far from complete. The data from our study provide a set of candidate proteins that hold the lariat-intron intermediate in C complex. Furthermore, they suggest a measure of stability between interaction partners for several spliceosome proteins at this stage of spliceosome progression. For example, SF3b's association with the lariat-intron intermediate appears to be stronger than its interactions with U2 snRNA. With NTC associated proteins, our data limit the number of potential interaction partners that stabilize their association with the lariat-intron intermediate. To date, very little is known about how these proteins incorporate into the spliceosome, and our data suggest that at this stage of the spliceosome some NTC components interact more intimately with this core lariat-intron complex than with other NTC members. This may be a reflection of the orientation of the NTC and its associated proteins relative to the lariat-intron intermediate and/or of a change in NTC conformation in C complex. Our data also suggest intriguing potential for interactions of NTC and its associated proteins with SF3b and/or other proteins that we identified in the stable lariat-intron intermediate complex. Future studies using the approach that we present here will certainly shed further clues for solving the complicated structural puzzle that spliceosome with its many components presents.

## Methods

### Pre-mRNA splicing substrates

All pre-mRNA splicing substrates are a derivative of the Adenovirus Major Late (AdML) transcript as a splicing substrate [25] that contains an AG→GG 3′ splice site mutation [26]. The substrate was tagged with three MS2 phage coat protein-binding sites in either the intron or 5′ exon for affinity purification in conjunction with a fusion of MS2 protein to maltose binding protein (MS2∶MBP).

### C complex purification

As previously described [14] C complex spliceosomes were assembled in an in vitro splicing reaction after which excess pre-mRNA not incorporated into the spliceosome was digested by endogeneous RNAse H by adding two DNA oligo nucleotides complementary to the region 6–28 nt upstream of the 5′ splice site. The complexes were isolated by size exclusion followed by affinity capture on amylose beads in 20 mM Tris, pH 7.9, 150 mM KCl, 0.5 mM EDTA (SCB1). Native C complexes were eluted from amylose beads using 10 mM maltose in SCB1, as previously described [14]. To isolate urea-washed complexes, the amylose beads were incubated in 3M urea for 5 minutes at room temperature. After collecting the resulting supernatant, the beads were submitted to regular elution with maltose.

### Glycerol gradient analyses

Native C complex or C complex incubated with 3M urea for 30 min at room temperature was centrifuged on a 600 µL linear 10–30% glycerol gradient prepared in SCB1 at 38,000 rpm for 2 h 30 min at 4°C using a Beckman SW 55 Ti rotor with tube adaptors. Fifteen 40 µL fractions were collected from the top to the bottom of the gradient. RNA from each fraction was extracted with phenol/chloroform, ethanol precipitated and separated on a 15% denaturing acrylamide gel, which was visualized with a phosphorimager (Molecular Dynamics) and quantified with ImageQuant software (Molecular Dynamics).

### Northern blots

For northern blots, 80 fmol of purified complexes and an aliquot of HeLa nuclear extract were separated on a denaturing 10% polyacrylamide gel, transferred to a nylon membrane (Immobilon, Millipore), UV crosslinked, and hybridized with ^32^P-labeled probes complementary to the U snRNAs [27].

### Western blots

80 fmol of purified samples along with an aliquot of HeLa nuclear extract were separated on a 10% SDS-PAGE, transferred to a nitrocellulose membrane and probed with either anti-SF3b130 or anti-PRPF8 (Proteintech). Following incubation with anti-IgG rabbit (Santa Cruz Biotech) the membrane was developed using the Super Sensitive Femto Kit (Pierce).

### Mass spectrometry

For peptide sequence analysis by mass spectrometry, approximately 500 fmol of purified complexes were separated by 10% SDS-PAGE and stained with Coomassie-G. The gel lane was cut into 8–10 sections, and each section subjected to in-gel tryptic digestion. Proteins were reduced with 10 mM DTT for one hour at 55C, then free sulfhydryls were alkylated using iodoacetamide for one hour, after which proteins were digested overnight with 150 ng Promega modified trypsin.

Samples were analyzed using two different mass spectrometry platforms, and then results were combined. Samples were analyzed by LC-MS/MS using a Thermo LTQ-Orbitrap and a MDS Sciex/Applied Biosystems QSTAR XL. One hour reverse-phase chromatography separations were carried out using an Eksigent nano-1D HPLC system attached to each mass spectrometer. Peptides were selected for fragmentation analysis as they eluted in a data-dependent fashion, and dynamic exclusion was employed to prevent repeated analysis of the same components.

Data was searched using Protein Prospector developmental version 4.25.4 (which was functionally similar to version 5.0 [28]) against the UniprotKB protein database downloaded on February 21^st^ 2007. For Orbitrap data, precursor and fragment mass tolerances of 15 ppm and 0.6 Da respectively were allowed, and for QSTAR data a precursor mass tolerance of 100 ppm and fragment mass tolerance of 300 ppm was considered. Cysteines were assumed to be carbamidomethylated, and methionine oxidation, protein N-terminal acetylation and pyroglutamate formation from N-terminal glutamine residues were all considered as possible modifications. A maximum expectation value of 0.1 was employed as a threshold for peptide identifications.

## Supporting Information

Figure S1 **Glycerol gradient profile of C complex spliceosomes treated with 1M urea.** The image shows denaturing gel analysis of RNA from linear (10–30%) glycerol gradient. Fractions numbers from the top to the bottom of the gradient are indicated. Splicing intermediates are indicated on the left. From top to bottom: lariat-intron intermediate, pre-mRNA, digested pre-mRNA and 5′ exon.
(TIF)
