# Supplementary figures and images for: Breaking Up the C Complex Spliceosome Shows Stable Association of Proteins with the Lariat Intron Intermediate
Source: PLoS One. 2011 Apr 19;6(4):e19061. doi: 10.1371/journal.pone.0019061 (PMC3079748; doi:10.1371/journal.pone.0019061)

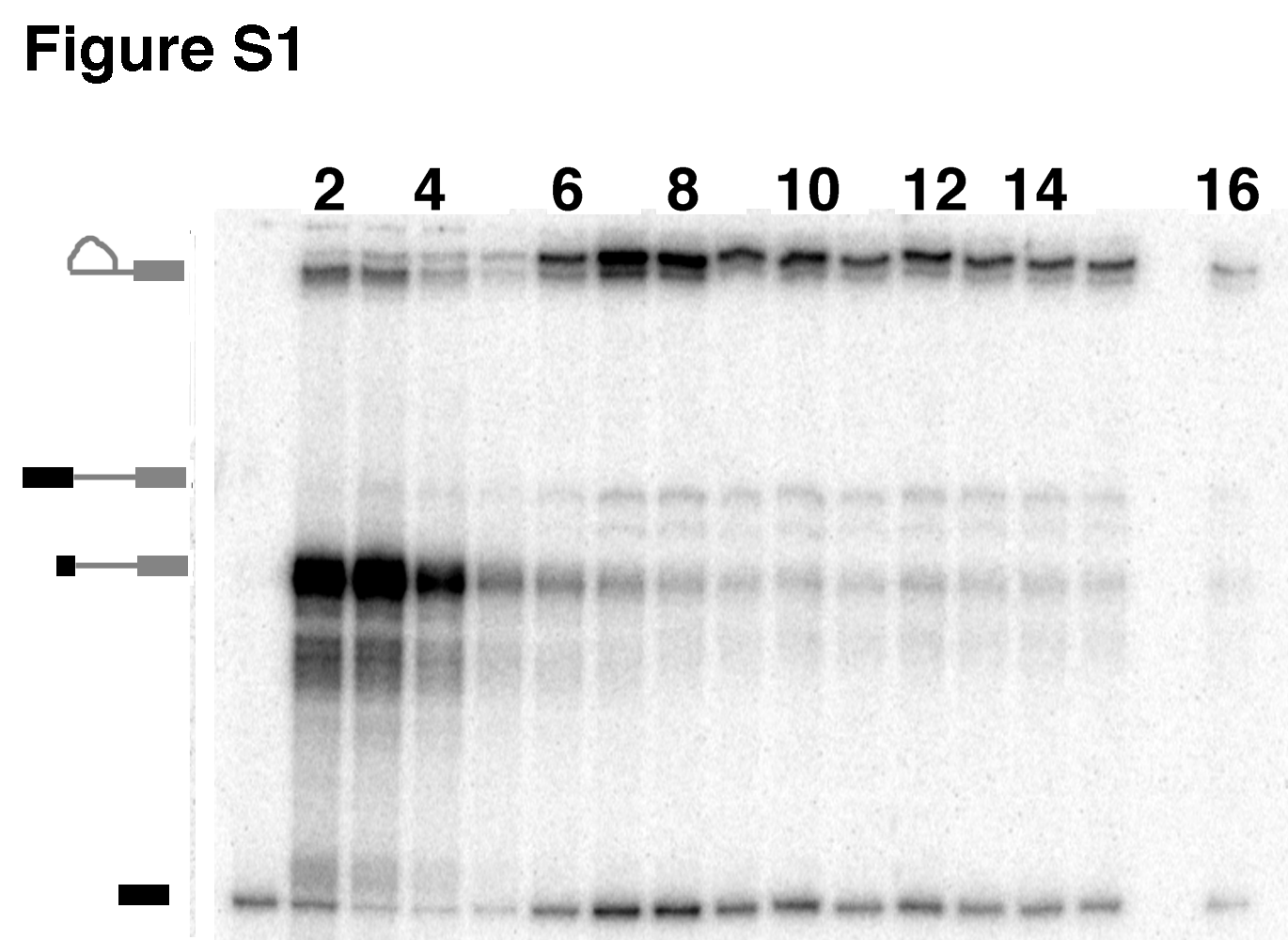

Supplement: Figure S1 — Glycerol gradient profile of C complex spliceosomes treated with 1M urea. The image shows denaturing gel analysis of RNA from linear (10–30%) glycerol gradient. Fractions numbers from the top to the bottom of the gradient are indicated. Splicing intermediates are indicated on the left. From top to bottom: lariat-intron intermediate, pre-mRNA, digested pre-mRNA and 5′ exon. (TIF) [file pone.0019061.s001.tif]
